# Afrina barna-like Virus, a Novel Virus Associated with *Afrina sporoboliae*, the Drop Seed Gall-Forming Nematode

**DOI:** 10.3390/v17081032

**Published:** 2025-07-23

**Authors:** Edison Reyes-Proaño, Anna M. Griffin, Aida Duarte, Hongyan Sheng, Brenda K. Schroeder, Timothy D. Murray, Alexander V. Karasev

**Affiliations:** 1 Department of Entomology, Plant Pathology and Nematology, University of Idaho, Moscow, ID 83844, USA; edison@uidaho.edu (E.R.-P.); grif2800@vandals.uidaho.edu (A.M.G.); bschroeder@uidaho.edu (B.K.S.); 2 Plant Pathogen Confirmatory Diagnostics Laboratory, USDA-APHIS-PPQ, Laurel, MD 20708, USA; aida.damasoduarte@usda.gov; 3 Department of Entomology and Plant Pathology, North Carolina State University, Raleigh, NC 27607, USA; 4 Department of Plant Pathology, Washington State University, Pullman, WA 99164, USA; hsheng@wsu.edu (H.S.); tim.murray@wsu.edu (T.D.M.)

**Keywords:** Barnavirus, nematode virus, *Afrina*

## Abstract

A novel barna-like virus was found to be associated with field-collected *Afrina sporoboliae* plant-parasitic nematodes. The positive-sense, single-stranded RNA genome of this virus, named Afrina barna-like virus (AfBLV), comprises 4020 nucleotides encoding four open reading frames (ORFs). ORF 1 encodes a protein product spanning a transmembrane, a peptidase, and VPg domains, whereas an overlapping ORF 2 encodes an RNA-dependent RNA polymerase (RdRP). ORF2 may be expressed via a −1 translational frameshift. In phylogenetic reconstructions, the RdRP of AfBLV was placed inside a separate clade of barna and barna-like viruses related to but distinct from the genera in the *Solemoviridae* and *Alvernaviridae* families, within the overall lineage of *Sobelivirales*. ORF 3 of AfBLV encodes a protein product of 206 amino acids (aa) long with homology to a putative protein encoded by a similarly positioned gene of an uncharacterized virus sequence identified previously as Barnaviridae sp. ORF 4 encodes a 161 aa protein with no significant similarities to sequences in the GenBank databases. AfBLV is the first barnavirus found in a nematode. Sequence comparisons of the AfBLV genome and genomes of other barna-like viruses suggested that a recombination event was involved in the evolution of AfBLV. Analyses of the phylogeny of RdRPs and genome organizations of barna-like and solemo-like viruses support the re-classification of *Barnavirus* and *Dinornavirus* genera as members of the *Solemoviridae* family.

## 1. Introduction

The formally accepted virus family *Barnaviridae* includes a single genus, *Barnavirus* [1], which contains only one species approved by the International Committee of Taxonomy of Viruses (ICTV)—mushroom bacilliform virus (MBV) [1]. The particles of MBV represent bacilliform-shaped, non-enveloped 19 × 50 nm virions that encapsidate a 4.0 kb positive-sense, single-stranded RNA genome coding for four major and three minor open reading frames (ORFs) [2]. ORF 1 codes for a protein of 22 kDa while ORFs 2, 3 and 4 encode for a serine protease, RNA-dependent RNA-Polymerase (RdRP), and a putative capsid protein (CP) of 73, 47 and 22-kDa, respectively [2]. Although the only approved member of the genus *Barnavirus*, MBV, has been associated with a fungal host, recent reports indicated that barna-like viruses may also be associated with other hosts, such as plants [3,4] and, possibly, mammals [5]. *Barnaviridae*, thus, represents a relatively poorly studied family of viruses, possibly in need of more research and phylogenetic refinement.

*Afrina sporoboliae* is a seed gall forming nematode able to induce seed galls in *Sporobolus cryptandrus*, a species of grass native to North America [6]. The life cycle of seed gall nematodes, such as *A. sporoboliae*, consists of the egg, four juvenile developmental stages, and the adult. The first two stages (J1 and J2) develop within the egg, and J2 hatch from the eggs inside seed galls, serving as the infective stage. During summer and fall, galls produced during the current season fall to the ground, and the nematodes enter the anhydrobiotic stage, where they can survive for many years. Galls rehydrate, releasing the nematodes onto the soil surface. The J2s migrate to nearby plants, where they penetrate tissue and induce the formation of new galls. Within galls, juvenile nematodes develop through two more stages (J3 and J4) into adults, which females produce several hundred eggs per gall [7]. *A. sporoboliae* is found in association with an undescribed *Rathayibacter* spp. and it is assumed that the nematode serves as the vector to deliver the bacterium to the plant inflorescence where it infects and produces seed galls [6,8]. This bacterium is related to the Select Agent *Rathayibacter toxicus*, a bacterium parasitic to grass hosts, which is a concern to the U.S. agriculture because it produces a tunicamycin toxin on ryegrass, causing Annual Ryegrass Toxicity when consumed by grazing livestock and is often fatal [9]. *A. sporoboliae* was initially found in Idaho in 2018 [6] and is the third *Afrina* species reported in North America. *A. sporoboliae* is currently the only *Afrina* species found on native plant species with no known disease management strategies. One of the possible biocontrol options for plant-parasitic nematode management is the use of viral pathogens; this option is considered in management of cyst nematodes affecting soybean and potato [10,11,12]. Multiple viruses from the families *Flaviviridae*, *Picornaviridae*, and *Rhabdoviridae* were found in populations of soybean cyst nematode [10] and potato cyst nematode [11,12,13].

To look for potential viruses circulating in *A. sporoboliae* populations, we subjected the nematode samples collected in Idaho to the RNAseq analysis using the total, ribodepleted RNA extracted from seed gall nematodes. In this study, we present the whole genome of Afrina barna-like virus (AfBLV), a new member of the *Barnaviridae* family that is associated with a population of drop seed gall-forming nematodes in Idaho.

## 2. Materials and Methods

### 2.1. Sample Origin, RNA Extraction, High-Throughput Sequencing, and Sequence Analysis

Seed galls containing *A. sporoboliae* nematodes were collected in 2022 from *S. cryptandrus* plants in Idaho County, Idaho, and stored at room temperature. The seed galls were wrapped in one layer of cheesecloth, soaked in 50 mL of sterile distilled water in a 250 mL beaker overnight to induce nematode hatching. The nematodes fell to the bottom of the beaker and were harvested through centrifugation (3000 rpm for 4 min), washed twice with 5 mL sterile distilled water; the J2 nematodes were surface sterilized with 5 mL of 0.5% sodium hypochlorite (3 min) followed by two washes with 5 mL sterile water for sodium hypochlorite removal (3000 rpm for 4 min) and stored at −80 °C (Ref. [6] and Duarte et al., unpublished). Total RNA was extracted separately from nine nematode samples (from A1 to I9), which consisted of approximately 20,000 juvenile J2′s nematodes, following the protocol described by Kud et al. [14], the RLT lysis buffer was supplemented with 2.5% polyvinylpyrrolidone (PVP) and 1.5% β-mercaptoethanol prior tissue disruption. Samples were subjected to DNase treatment using a DNase kit (QIAGEN, Germantown, MD, USA) and submitted for high-throughput sequencing (HTS) at GENEWIZ facilities (Azenta, South Plainfield, NJ, USA). After quality control, samples with an RNA Integrity Number (RIN) exceeding 6 were subjected to ribodepletion using the FastSelect rRNA Worm Kit (Qiagen, Hilden, Germany) prior to the complementary DNA (cDNA) library construction. The cDNA library was generated using NEBNext Ultra II RNA Library Preparation Kit for Illumina by following the manufacturer’s recommendations (NEB, Ipswich, MA, USA). The sequencing was accomplished using the NovaSeq X Plus platform in a 150 nt paired-ends format. The reads obtained through HTS were processed using a custom pipeline involving trimming, filtering, mapping and BLASTx analyses. Raw reads were trimmed and quality-filtered using Trimmomatic v0.39. Paired reads were mapped to the 30–1019090437_Afrina_sporoboliae_ERCC reference genome (Duarte et al., unpublished) and de novo assembled using SPAdes v3.15.3. Contigs were analyzed using BLASTn and BLASTx tools against a custom viral database retrieved from National Center for Biotechnology Information (NCBI) (https://www.ncbi.nlm.nih.gov/ (accessed on 4 April 2024)). Pair-wise comparisons of nucleotide and amino acid sequences were conducted using BLASTn and BLASTx programs provided by NCBI. Genome conceptual translation and ORF predictions were performed using Geneious Prime 2023.1.1. Identification of conserved protein domains was performed using the CDD program provided by the NCBI (https://www.ncbi.nlm.nih.gov/Structure/cdd/wrpsb.cgi (accessed on 23 March 2025)) and HHPred [15]. Additionally, the conserved domain analysis was supplemented with a prediction for transmembrane domains using TMHMM (https://services.healthtech.dtu.dk/services/TMHMM-2.0/ (accessed on 23 March 2025)).

### 2.2. RT-PCR Validation, Sanger Sequencing of the Genome, and Phylogenetic Analysis

To validate the sequences found in the HTS data, two sets of specific primers (AfBLV-4F/4R and AfBLV-6F/6R) were designed (Supplementary Table S1). First strand cDNA and polymerase chain reaction (PCR) were synthesized from total RNA using SuperScript™ III Reverse Transcriptase (Thermo Fisher Scientific, Waltham, MA, USA) and GreenTaq DNA Polymerase (GenScript, Piscataway, NJ, USA), following the protocol described by Reyes-Proaño et al. [16]. Following the visualization of amplicons on 1.2% agarose gels, PCR products were treated using ExoSAP-It (Applied Biosystems, Waltham, MA, USA) as described by Green et al. [17] and submitted for Sanger sequencing to Elim Biopharmaceuticals (Hayward, CA, USA). The full-length genome of AfBLV was re-sequenced using overlapping PCR products amplified with specific primers (Supplementary Table S1). The cDNA of the 5′- and 3′-terminal regions were acquired using primers AfBLV_5-1 and AfBLV_3-3, respectively, using the 5′/3′ rapid amplification of cDNA ends (RACE) Kit (Roche, Indianapolis, IN, USA) following manufacturer’s instructions. The complete genome of AfBLV was assembled with Geneious Prime 2023.1.1. (Biomatters, Inc., Boston, MA, USA). Phylogenetic inferences were conducted using amino acid sequences from the RdRP coding sequence of virus species accepted by the ICTV as members of the order *Sobelivirales*. Sequences corresponding to RdRP were aligned using ClustalOmega3 and the best amino acid substitution model was determined using MEGAX. Phylogenetic tree was obtained using MEGAX using the LG+G+I+F as the best fit protein model with 1000 bootstrap replicates [18].

## 3. Results

### 3.1. High-Throughput Sequencing Analysis of the Nematode Samples

**Figure 1:**
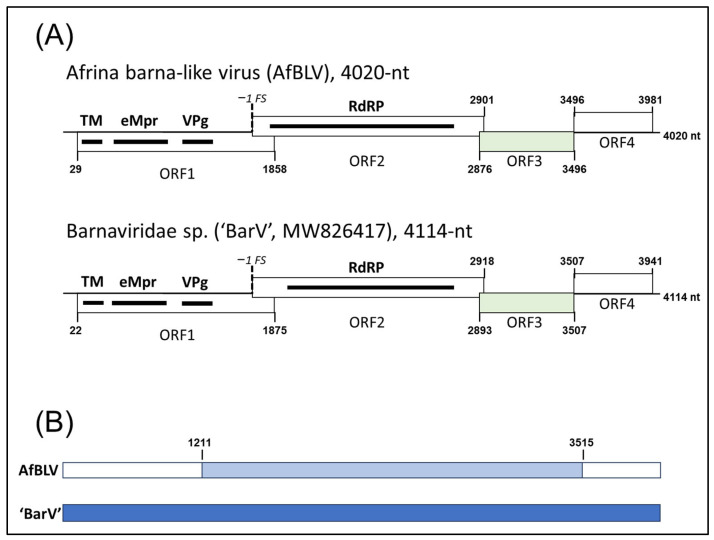
Schematic representation of the Afrina barna-like virus (AfBLV) genome (GenBank accession PV941960) side-by-side with Barnaviridae sp. virus (‘BarV’, MW826417). (**A**) AfBLV and BarV genomes encode four open reading frames (ORFs), with conserved protein domains designated as thick black lines; TM = transmembrane domain, eMpr = V8-like Glu-specific endopeptidase, VPg = viral protein genome-linked, RdRP = RNA dependent RNA polymerase. ORFs 3 for both viruses are shaded in light green as an indication of a significant amino acid sequence similarity. (**B**) Schematic diagram of a possible recombinant structure of AfBLV: BarV genome is arbitrarily set as a ‘parental’ genome (blue color), with ABLV genome containing a ca. 2.3 kb segment (light blue color) with 75% nucleotide sequence identity to the ‘parental’ BarV genome; the rest of the ABLV genome displays no significant similarity to BarV at the nucleotide sequence level (white color).

High-throughput sequencing of each of the nine field samples of *A. sporoboliae* produced between 50 and 84 million paired-end raw reads, and, after removal of reads mapped to the nematode genome (between 78.39 and 89.76%), allowed to assemble de novo between 22,846 and 30,048 contigs over 500 nt in length (Table 1). Initial bioinformatics analysis revealed the presence of a single, ca. 4.0 kb virus-related contig in sample A1 encoding four open reading frames (ORFs) (Figure 1A). Given the number of ORFs, their organization, and presence of characteristic conserved domains with visible similarity to barna- and barna-like sequences (see below), it was assumed that the identified contig represented complete or nearly complete genome of a new barna-like virus from *A. sporoboliae* and it was named Afrina barna-like virus (AfBLV). To exclude the possibility of AfBLV originating from *S. cryptandrus* or contaminating fungal organisms, the reads were mapped against fungal and plant databases retrieved from the NCBI. The number of reads mapped against fungal genomes varied between 0.0015% and 0.0060%, depending on an individual sample, significantly lower than the number of reads mapped to the AfBLV genome (Table 1), excluding the fungal origin of AfBLV. The number of reads mapped against a *Sporobolus maritimus* plant genome (GCA_965119375.1) varied between 0.0395% and 0.0921%, depending on an individual sample, and was significantly lower than the number of reads mapped to the AfBLV genome (Table 1), excluding the plant origin of AfBLV.

### 3.2. Sanger Sequencing of the Afrina barna-like Virus Genome

The presence of the AfBLV in all nine samples of *A. sporoboliae* was confirmed using RT-PCR with subsequent Sanger sequencing of the PCR products (Table 1). The entire genome of AfBLV was Sanger sequenced from the A1 sample, and this Sanger-derived sequence was found to be 99.1% identical to the sequence obtained via HTS. The 5′ and 3′-termini of the AfBLV genome were amplified using the RACE approach, as described in the Materials and Methods Section, and the complete genome of AfBLV was found to be 4020 nt long (Figure 1A) with the 5′-untranslated region (UTR) being 28 nt, and the 3′-UTR being 39 nt. The complete genome of AfBLV was assembled from the Sanger-derived sequence reads and deposited in the GenBank database under the accession number PV941960. In pair-wise comparisons, a central ca. 2.3 kb section of the AfBLV genome exhibited 75% nucleotide sequence identity to the uncharacterized Barnaviridae sp. isolate XZS182276 (‘BarV’, MW826417), while the 5′-terminal 1.2 kb and 3′-terminal 0.5 kb of AfBLV did not show significant nucleotide sequence similarity to either the ‘BarV’ sequence or to any other sequences in GenBank (see Figure 1B). This nucleotide sequence identity level translated into relatively high amino acid similarity levels for protein products encoded by ORFs 1–3 of AfBLV and ‘BarV’ sequence and to lesser extent for other barna-like virus protein products (Table 2). AfBLV, thus, represents the first barna-like virus found in a nematode host.

ORF 1 coded for a protein of 611 amino acids (aa) with a molecular weight of 66.1 kDa, containing a conserved V8-like Glu-specific endopeptidase domain (eMpr) identified by the CD-search program. This 66 kDa protein exhibited similarities with several peptidase-like proteins of barna and solemovirids, in particular, with one barna-related virus sequence obtained from metagenomic studies of rat feces [Barnaviridae sp. isolate XZS182276 (‘BarV’); MW826417.1] identified as the closest match in BLASTx searches (64.9% identity, 90% coverage). A transmembrane domain (TM) flanked by phenylalanine in position 4 and valine in position 29 was also identified in the protein product encoded by ORF1 (Figure 1A). Additionally, a HHPred search of this 66 kDa protein detected a region of 88 amino acids resembling the viral-protein-genome-linked protein (VPg) of sobemoviruses. The arrangement of these domains in the genome of AfBLV (Figure 1) resembled those found in the ORF 1-encoded proteins of poinsettia latent virus (AJ867490, *Polemovirus*), potato leafroll virus (D13954, *Polerovirus*) pea enation mosaic virus (L04573, *Enamovirus*) and in the ORF 2a-encoded protein of southern bean mosaic virus (DQ875594, *Sobemovirus*) [19].

ORF 2 of AfBLV coded for a 554 aa protein of 61.8 kDa with an easily identifiable RdRP domain, matching the RdRP-encoding protein of the same ‘BarV’ sequence (MW826417.1) with 81.9% identity at the aa level and 62% coverage. The presence of a putative heptameric slippery signal _1231_UUUAAAC_1237_, right before ORF 2, suggested a −1 ribosomal frameshift as an expression strategy for ORF 2. ORF 3 coded for a putative protein of 212-aa and 23.6 kDa molecular weight; this protein shared the 73.3% aa sequence identity with the protein encoded by the ORF 3 of the same ‘BarV’ sequence (MW826417.1) with 83% coverage. In contrast, the downstream ORF 4 encoded a 162-aa protein of 17.8 kDa that produced no matches in the GenBank database when either BLASTx or BLASTp were used for analyses.

### 3.3. Afrina barna-like Virus Is a New Species in the Genus Barnavirus

The genome of AfBLV had a genomic organization similar to ‘BarV’ (MW826417.1) and to ApBLV-1 (MN386956.1) (Figure 1). Given the low similarities at the nucleotide and protein sequence levels and genetic relationships to species in the genus *Barnavirus*, we propose AfBLV to be a new member of this genus associated with *A. sporoboliae*, the drop seed gall forming nematodes. Although no formal threshold for species demarcation exists in the family *Barnaviridae* [1], the 75% nucleotide sequence identity lies at the very border of the species demarcation in other virus families, such as *Potyviridae* [20]. However, since this relatively distant similarity level corresponds to less than 60% of the AfBLV genome (Figure 1B), we feel confident that AfBLV represents a new virus species within the family *Barnaviridae*.

**Figure 2:**
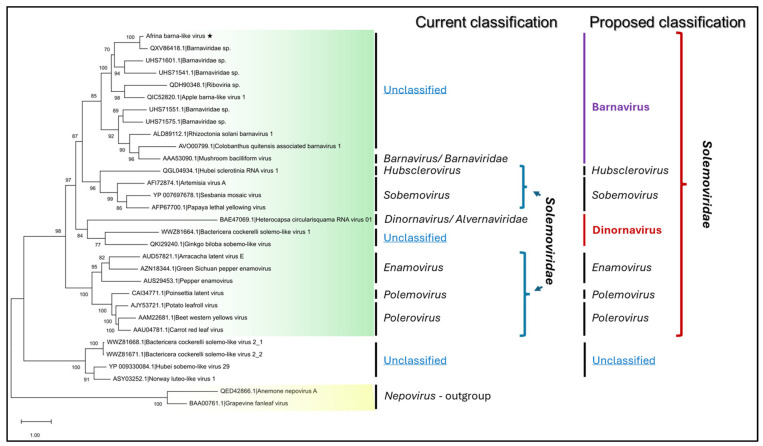
Maximum likelihood phylogenetic consensus tree based on the predicted amino acid sequence of the RdRP depicting the genetic association of AfBLV (PV941960) to other species in the order *Sobelivirales*. ICTV accepted families are highlighted in colored boxes as follows. Green: virus species belonging to the approved families *Solemoviridae*, *Barnaviridae*, and *Alvernaviridae*. Yellow: outgroup sequences of anemone nepovirus A (QED42866.1) and grapevine fanleaf virus (BAA00761.1) from the family *Secoviridae* were used for phylogenetic tree construction. Bootstrap values are represented at each node. Two panels to the right of the tree present the positions of the analyzed virus RdRP sequences in a current classification of genera from the *Solemoviridae, Barnaviridae,* and *Alvernaviridae* families, proposing amendments to this classification. Genera and families comprising virus species currently approved by ICTV are italicized; new genera Barnavirus (purple) and Dinornavirus (red) are highlighted in bold. Clades and lineages not classified in the current or proposed classifications are designated as “unclassified”.

Since BLASTx searches identified barna-like virus sequences as the closest matches to AfBLV, phylogenetic relationship was inferred with RdRPs of all known barna and barna-like sequences available in the GenBank database, along with select sequences from the families *Solemoviridae* and *Alvernaviridae*; additional unclassified solemo-like virus sequences found recently in arthropods [21,22,23] were also included in the analysis (Figure 2). The resulting phylogenetic tree placed AfBLV in a well-supported clade containing a sole approved member of the family *Barnaviridae*, MBV, plus apple barna-like virus 1 (ApBLV-1) from apple leaf samples (QIC52820), and multiple viral sequences obtained from soil sediment metagenomic studies (see Figure 2). It is conceivable to propose that AfBLV, ApBLV-1, and related sequences from soil sediment metagenomics data be included into the genus *Barnavirus* along with the approved species, MBV. This same phylogenetic analysis placed the barna and barna-like virus clade close to viruses from the families *Solemoviridae* and *Alvernaviridae* within the order *Solemovirales*, and close to a separate clade of unclassified solemo-like viruses found in insects [21,23] and in ticks [22] (see Figure 2).

## 4. Discussion

The family *Solemoviridae* currently comprises four approved genera, *Sobemovirus*, *Polemovirus*, *Polerovirus*, *Enamovirus*, and *Hubsclerovirus*. These are viruses with positive-sense, single-strand RNA genome of 4–6 kb that are encapsidated with CP into icosahedral virions (20–34 nm in diameter); the great majority of solemovirids infect plants [19], with the exception of the genus *Hubsclerovirus* comprising fungal solemovirids [24]. There are two families, *Barnaviridae*, described in the introduction, and *Alvernaviridae* that are closely related to *Solemoviridae*.

The ICTV-approved family *Alvernaviridae* includes a single genus, *Dinornavirus* [25], which contains only one species approved by the ICTV, Heterocapsa circularisquama RNA virus 01 (HcRNAV01) [25]. The particles of HcRNAV01 are polyhedral, approximately 30 nm in diameter and encapsidate a 4.4 kb positive-sense, single-stranded RNA genome coding for two ORFs [26]. ORF1 codes for a protein with serine protease and RdRP domains, and ORF2 encodes the CP [26]. The only approved member of the genus *Dinornavirus*, HcRNAV01, has been associated with a dinoflagellate (*Heterocapsa circularisquama*) as a host. This phylogenetic analysis indicates that at least two other virus sequences, Bactericera cockerelli solemo-like virus 1 (BcSLV-1) [21] from an insect host, and Gingko biloba sobemo-like virus 1 (GbSLV) [27] from a plant host, are closely related to dinornavirids (Figure 2), and may be placed in the same genus *Dinornavirus*. Similarly to the family *Barnaviridae*, the family *Alvernaviridae* appears to be a relatively poorly studied family of viruses, and in need of phylogenetic refinement.

Careful inspection of the phylogeny of virus RdRP sequences from ICTV-approved families *Barnaviridae*, *Alvernaviridae*, and *Solemoviridae* (Figure 2) prompted us to propose some taxonomic changes within the family *Solemoviridae* to improve and simplify classification of these related viruses. Specifically, two ICTV-approved genera, *Barnavirus* and *Dinornavirus* seem to represent distinct clades inside the family *Solemoviridae* (Figure 2) and could be considered for inclusion in the family *Solemoviridae*. The two old family names, *Barnaviridae* and *Alvernaviridae* could be retired and abolished due to re-location of the single genus in each of the two families into *Solemoviridae*. The arrangement of the conserved motifs in replication-associated proteins, the −1 ribosomal frame-shifting strategy for the expression of the RdRP protein, and the monophyletic lineage in the RdRP-based phylogeny, all support the expansion of the family *Solemoviridae* to include the genera *Barnavirus* and *Dinornavirus*. These suggestions to rearrange the family *Solemoviridae* are summarized in Figure 2.

Recombination is one of the main forces driving evolution of positive-strand RNA viruses [28,29,30]. Here, we present the first evidence of recombination involved in the evolution of barnaviruses. The genome of the newly found AfBLV is apparently built from three segments, with a central 2.3 kb section of the genome exhibiting 75% nucleotide sequence identity to the genome of an uncharacterized Barnaviridae sp. isolate XZS182276 (‘BarV’, MW826417); and the 1.2 kb 5′-terminal section and the 0.5 kb 3′-terminal sections of the genome coming from a barnavirus only distantly related to AfBLV (no significant nt similarity) (Figure 1B). This central section of the genome includes the entire ORFs 2 and 3 of AfBLV, encoding RdRP and an unknown protein (Figure 1A,B). It is tempting to speculate that ORFs 2 and 3 of barna-like viruses may be responsible for more conserved virus functions, e.g., virus replication, while ORFs 1 and 4 could represent virus functions specific for different host species supporting barnavirus replication. It is difficult to speculate about the host specificity of BarV though, since limited information in the GenBank annotation file specifies rat feces as the origin of the virus sample and no publication is available, but a nematode could be one possible source.

Recently, several reports based on metagenomics analyses, indicated that barna-like viruses may be associated not only with fungi, but also with other hosts, such as plants [3,4] and, possibly, mammals [5]. The discovery of AfBLV isolated from *A. sporoboliae* expands the host range of barnaviruses even further and now includes at least one phylum of invertebrate animals. Different viruses were found in populations of free-living [31,32,33] and parasitic [23,34], including plant-parasitic, nematodes [10,11,12,13,35,36,37,38]; however, to the best of our knowledge, AfBLV is the first barna-like virus found in a nematode species. The impact of this virus on the nematode is unknown and further work is needed to assess the effect of the virus on the nematode’s life cycle; nevertheless, the discovery of AfBLV could potentially expand the biocontrol strategies to manage the *Afrina sporoboliae* nematodes.

## Figures and Tables

**Table 1:** Summary of the high-throughput sequencing and Sanger-sequencing data generated for all nine *Afrina sporoboliae* samples.

Sample	Raw Reads	Post-Trimming Reads	Contigs de novo Assembled	AfBLV ^1^	# of Reads Assembled to the AfBLV Genome	# of Reads (and %) Assembled to Fungi Genomes	# of reads (and %) Mapped to *A. sporoboliae*	# of Reads (and %) Mapped to *S. maritimus*
A1	53,376,244	34,452,026	28,061	+	157,584	2074 (0.0060%)	30,031,802 (87.17%)	21,627 (0.0628%)
B2	62,948,370	44,743,366	30,048	+	191,913	2037 (0.0046%)	40,161,846 (89.76%)	24,340 (0.0544%)
C3	58,966,450	36,426,824	28,292	+	140,764	1491 (0.0041%)	30,341,392 (83.29%)	21,435 (0.0588%)
D4	50,944,370	32,743,486	28,154	+	187,638	1973 (0.0060%)	28,728,534 (87.74%)	30,158 (0.0921%)
E5	56,670,030	36,513,632	27,370	+	152,651	1601 (0.0044%)	30,280,354 (82.93%)	24,057 (0.0659%)
F6	50,546,932	29,786,438	22,846	+	79,619	891 (0.0030%)	23,349,988 (78.39%)	14,400 (0.0483%)
G7	84,220,492	49,407,814	23,567	+	86,006	2126 (0.0043%)	38,836,542 (78.60%)	24,243 (0.0491%)
H8	83,110,782	49,872,072	24,216	+	85,055	1866 (0.0037%)	39,912,720 (80.03%)	23,123 (0.0464%)
I9	84,308,846	50,441,592	25,032	+	104,713	777 (0.0015%)	40,338,140 (79.97%)	19,926 (0.0395%)

^1^ AfBLV = Afrina barna-like virus: + designates virus-positive status confirmed by RT-PCR and Sanger sequencing.

**Table 2:** Pair-wise comparisons between sequences of genomes, genes, and protein products of Afrina barna-like virus (AfBLV) and phylogenetically related barna-like virus sequences as percentages at the nucleotide (nt) and amino acid (aa) levels.

		‘BarV’ MW826417		Barnaviridae sp. MZ218180		Barnaviridae sp. MZ218210 ^1^	
		nt	aa	nt	aa	nt	aa
AfBLV	ORF 1 ^2^	66.5	61.2	NS	NS	NS	NS
	ORF 2	75.0	74.2	NS	35.3	51.3	49.9
	ORF 3	64.2	69.8	NS	NS	-	-
	ORF 4	NS ^3^	NS	NS	NS	-	-
	Genome	64.7	-	NS	-	NS	-

^1^ Partial sequence; ^2^ ORF = open reading frame; ^3^ NS = not statistically significant.

## Data Availability

The AfBLV genome sequence reported is available in GenBank under accession number PV941960.

## Supplementary Materials

The following supporting information can be downloaded at: https://www.mdpi.com/article/10.3390/v17081032/s1, Table S1: Primers used for genome resequencing, detection and 5′/3′ acquisition of AfBLV.
