# Supplementary material for: Afrina barna-like Virus, a Novel Virus Associated with Afrina sporoboliae, the Drop Seed Gall-Forming Nematode
Source: Viruses. 2025 Jul 23;17(8):1032. doi: 10.3390/v17081032 (PMC12390465; doi:10.3390/v17081032)
Supplement: Supplementary file 1 [file viruses-17-01032-s001.zip › viruses-3737599-supplementary.pdf]

Supplementary table S1: Primers used for genome resequencing, detection and 5'/3' acquisition of AfBLV.

Stock_ID	Amplicon size (bp)	Sequence 5' - 3'	Purpose	Annealing Temperature (°C)
AfBLV-1F	628	CCTACCTCCTGACCACTCTGG	Resequencing	60
AfBLV-1R		GACTACACGACCAACAACTGGTG		
AfBLV-2F	671	GCTCGAGAACCTCCCTGTCC	Resequencing	60
AfBLV-2R		AATGTGGTCGGAGACTCGC		
AfBLV-3F	899	CACCGCCGGCTCCTACTAT	Resequencing	60
AfBLV-3R		AATGAGCTGCTGTGGGCTC		
AfBLV-4F	777	GGAGCGCATTCCACAAGG	Resequencing/Detection	60
AfBLV-4R		CCGTGGAAGACAGTCACGTC		
AfBLV-5F	711	TGTACTACGACCCAAAGCTCATG	Resequencing	60
AfBLV-5R		CGTTCACGTCGTCAAAATACG		
AfBLV-6F	679	ACAGGGGATCTCTTCACCG	Resequencing/Detection	60
AfBLV-6R		GGACAGGTACTCAACAGCGC		
AfBLV-7F	372	GTCTTTGTGGCCGAGTGGTTC	Resequencing	60
AfBLV-7R		CAACTAGGCTCTCCTCCAGCG		
AfBLV_5-1	-	CGAACACCACCAAATATAGCTGC	RACE	60
AfBLV_5-2	-	TACAGACTAAACATGTAGGCCG	RACE	60
AfBLV_5-3	-	GCCACCAGGAAGAAAAGAGG	RACE	60
AfBLV_3-1	-	GCTGGAGGAGAGCCTAGTTGAT	RACE	60
AfBLV_3-2	-	ATGGAGTGATGGTTCATCCGTT	RACE	60
AfBLV_3-3	-	GCGCTGTTGAGTACCTGTCC	RACE	60
